# A phase II study of belumosudil for chronic graft-versus-host disease in patients who failed at least one line of systemic therapy in China

**DOI:** 10.1186/s12916-024-03348-5

**Published:** 2024-03-26

**Authors:** Ying Wang, Depei Wu, Xiang Zhang, Yuhua Li, Yanjie He, Qifa Liu, Li Xuan, Zhenyu Li, Kunming Qi, Yuqian Sun, Shunqing Wang, Wenjian Mo, Lei Gao, Ye Hua, Yu Wang, Ying Zhang

**Affiliations:** 1 The First Affiliated Hospital of Soochow University, No.188, Shizi Street, Gusu District, Suzhou, 215006 China; 2 Zhujiang Hospital, Southern Medical University, Guangzhou, China; 3 Nanfang Hospital, Guangzhou, Guangdong China; 4 The Affiliated Hospital of Xuzhou Medical University, Xuzhou, China; 5 Peking University People’s Hospital, Beijing, China; 6 Guangzhou First People’s Hospital, Guangzhou, China; 7 Xinqiao Hospital of Army Medical University, Chongqing, China; 8 BioNova Pharmaceuticals (Shanghai) Limited, Shanghai, China

**Keywords:** Belumosudil, cGVHD, ROCK2 inhibitor, ROCK, China, Efficacy, Safety

## Abstract

**Background:**

Chronic graft-versus-host disease (cGVHD) is an immune-related disorder that is the most common complication post-allogenic hematopoietic stem cell transplant. Corticosteroids with or without calcineurin inhibitors (CNIs) remain the mainstay of cGVHD treatment for first-line therapy. However, for many patients, cGVHD symptoms cannot be effectively managed and thus require second-line therapy. Currently, there is no approved treatment for second-line cGVHD treatment in China. In this study, belumosudil, a highly selective and potent rho-associated coiled-coil-containing protein kinase-2 inhibitor demonstrated to be effective for cGVHD in the United States and other Western countries, is investigated in patients with cGVHD in China for its overall benefit–risk balance.

**Methods:**

This multicenter, open-label phase II study evaluated the safety, efficacy, and pharmacokinetics of oral belumosudil 200 mg once daily in cGVHD patients who had been treated with at least one line of systemic therapy in China. The primary endpoint was overall response rate (ORR); each individual patient’s response was assessed by the investigator using the 2014 National Institutes of Health consensus criteria. Secondary endpoints were duration of response (DOR), time to response (TTR), changes in Lee Symptom Scale (LSS) score, organ response rate, corticosteroid dose change, CNI dose change, failure-free survival, time-to-next-treatment, overall survival, and safety.

**Results:**

Thirty patients were enrolled in the study with a median follow-up time of 12.9 months. ORR was 73.3% (95% confidence interval: 54.1–87.7%) and all responders achieved partial response. Median DOR among responders was not reached and median TTR was 4.3 weeks (range: 3.9–48.1). Fifteen patients (50.0%) achieved clinically meaningful response in terms of reduction in LSS score by ≥ 7 points from baseline. Corticosteroid and CNI dose reductions were reported in 56.7% (17/30) and 35.0% (7/20) of patients, respectively. Most treatment-emergent adverse events (TEAEs) were mild to moderate in severity, with 11 patients (36.7%) experiencing grade ≥ 3 TEAEs. The most common grade ≥ 3 TEAE was pneumonia (*n* = 5, 16.7%).

**Conclusions:**

Belumosudil treatment demonstrated a favorable benefit–risk balance in treating cGVHD patients who previously have had standard corticosteroid therapy in China where approved second-line setting is absent.

**Trial registration:**

ClinicalTrials.gov identifier NCT04930562.

**Supplementary Information:**

The online version contains supplementary material available at 10.1186/s12916-024-03348-5.

## Background

Chronic graft-versus-host disease (cGVHD), an immune-related disorder characterized by inflammation and fibrosis, is a common cause of morbidity and non-relapse mortality following allogenic hematopoietic stem cell transplant (allo-HSCT) [1–3]. The incidence of cGVHD is estimated to be 30–70% among patients who have received allo-HSCT [1]. In China, the prevalence of cGVHD among post–allo-HSCT patients is approximately 53% [4]. cGVHD can present with multiorgan involvement, affecting patients’ quality of life and long-term survival [2]. Patients with cGVHD typically require prolonged treatment or even hospitalization, with some severe complications even requiring life-saving treatments [3].

Systemic corticosteroids are the mainstay of cGVHD treatment in the first-line setting either given alone or in combination with other agents [5]. However, 50–60% of patients will require second-line treatment within 2 years, due to the toxicity of corticosteroids with long-term use or lack of efficacy especially among high-risk patients [5]. Currently, there is no approved second-line treatment for cGVHD in China. Therefore, there is clearly an unmet medical need for cGVHD treatment after systemic corticosteroid use.

Belumosudil is a novel first-in-class selective rho-associated, coiled-coil-containing protein kinase 2 (ROCK2) inhibitor that works by restoring type 17 T helper/regulatory T (Treg) cell balance and reducing fibrosis. In preclinical studies, belumosudil inhibited signal transducer and activator of transcription 3 phosphorylation, consequently downregulating the secretion of interleukin-21 and interleukin-17, shifting the type 17 T helper/Treg cell balance toward the Treg phenotype [6, 7]. Targeting ROCK2 also increases signal transducer and activator of transcription 5 phosphorylation, causing the upregulation of Treg cells that induces an immunomodulatory effect and reduces inflammation [7]. ROCK2 inhibition by belumosudil additionally downregulates the fibrosis process by suppressing profibrotic gene expression and transforming growth factor beta signaling [8]. This prevents the polymerization of G-actin to F-actin as well as decreasing myocardin-related transcription factor-mediated transcription, effectively reducing the differentiation of fibroblast to myofibroblast and collagen production, that otherwise are distinct manifestations of cGVHD [8]. This is demonstrated by the decreased collagen deposition in the lungs around the bronchioles and the delayed development of scleroderma in cGVHD animal models [9].

Belumosudil was first approved in the United States for the treatment of cGVHD in patients after failure of at least two prior lines of systemic therapy based on data from the phase II ROCKstar trial. In that study, the best overall response rate (ORR) was 76% in the overall population, and response status was sustained for a median duration of 54 weeks among the responders. Belumosudil was also proven to be well tolerated in this population, and its safety profile was consistent to that of post-transplant patients living with cGVHD [10, 11].

To further investigate the overall benefit–risk balance for belumosudil in patients with cGVHD in China, this multicenter, single-arm, open-label, phase II study was initiated in 2021. The final study results are presented here (ClinicalTrials.gov identifier: NCT04930562).

## Methods

### Study design and patients

This was a multicenter, single-arm, open-label phase II study evaluating the efficacy, safety, and pharmacokinetics of belumosudil in patients with cGVHD in China. Key eligibility criteria included patients: 1) aged ≥ 18 years who had received allo-HSCT; 2) with persistent manifestation of cGVHD that required systemic treatment; 3) who previously received at least one but not more than five lines of systemic therapy for cGVHD; and 4) who received corticosteroids at a stable dose for at least 2 weeks prior to screening. Patients were excluded if they had previously received an investigational systemic treatment for cGVHD (corticosteroids, calcineurin inhibitors [CNI], sirolimus, mycophenolate mofetil, methotrexate, and azathioprine were acceptable and patients must have been on a stable dose/regimen for ≥ 2 weeks prior to screening) within 28 days before study enrollment (unless there was a washout period of ≥ 28 days or five half-lives prior to enrollment), had a forced expiratory volume in 1 s ≤ 39% or pulmonary function score of 3 at screening, had histological relapse of their underlying malignancy or post-transplant lymphoproliferative disease at the time of screening, or were currently receiving ibrutinib. Full inclusion and exclusion criteria are in Additional file 1: Table S1.

All enrolled patients received oral belumosudil 200 mg once daily continuously in a 28-day cycle until cGVHD progression, intolerable toxicity, initiation of a new cGVHD therapy, recurrence of hematologic malignancy, loss to follow-up, withdrawal of consent, or death, whichever occurred first. Patients who did not achieve any response after 12 cycles and had no clinical benefit according to investigator’s judgement could withdraw from the study. Concomitant use of other cGVHD treatments (i.e., corticosteroids, CNIs, mycophenolate mofetil, sirolimus, methotrexate, and azathioprine), which started before screening, were allowed to continue during the study with the stable dose/regimen.

The study was conducted in accordance with the principles of the Declaration of Helsinki and Good Clinical Practice guidelines from the International Conference on Harmonisation, and local applicable regulatory requirements. All patients provided written informed consent prior to screening. The study protocol, any amendments, informed consent, and other documents provided to the participants were approved by the institutional review boards/independent ethics committee at the leading site (First Affiliated Hospital of Soochow University) and participating institutions.

### Study endpoints and assessment

The primary efficacy endpoint was investigator-assessed ORR (defined as complete response [CR] or partial response [PR]) based on the 2014 National Institutes of Health (NIH) consensus [12]. Secondary endpoints included duration of response (DOR), time to response (TTR), changes in Lee Symptom Scale (LSS) score, and organ response rate with organ-specific signs and symptoms of cGVHD scored according to the 2014 NIH cGVHD response criteria [12], corticosteroid dose change, CNI dose change, failure-free survival (FFS), time-to-next-treatment (TTNT), and overall survival (OS). DOR was defined as the time from the first assessment of CR or PR to the first detection of a lack of response. If the patient experienced progression from CR or PR to a lack of response more than once, the cumulative DOR was recorded. Response was assessed on day 1 of cycles 2–5 and every two cycles thereafter (day 1 of cycles 2, 3, 4, 5, 7, 9, 11, etc.). FFS was defined as the absence of new cGVHD systemic therapy, non-relapse mortality, and recurrent malignancy (i.e., underlying disease). The safety of belumosudil was evaluated by treatment-emergent adverse events (TEAEs) and serious adverse events (SAEs) coded using Medical Dictionary for Regulatory Activities version 25.1, 12-lead electrocardiograms, vital signs, and laboratory examinations. Blood samples were collected for drug metabolism and pharmacokinetic assays. The blood sampling schedule is presented in Additional file 1: Table S2.

### Statistical analysis

Sample size was not calculated for hypothesis testing using statistical assumptions. For the purpose of bridging the exposure and treatment effect of belumosudil in patients with cGVHD in China to those in the United States, this study was adequately powered with 30 patients. All statistical analyses were performed using SAS version 9.4 (SAS Institute, Cary, NC, USA).

The efficacy and safety analyses were conducted using the modified intent-to-treat (mITT) population, which was defined as all enrolled patients who received at least one dose of belumosudil. The ORR and 95% confidence interval (CI) were calculated based on the exact probability method of binomial distribution. Secondary efficacy endpoints were descriptively analyzed. The median and 95% CI were calculated for DOR, FFS, TTNT, and OS using the Kaplan–Meier method. Safety analysis was performed on the safety set, which comprised all patients who received at least one dose of belumosudil and had at least one safety assessment. The overall occurrence of adverse events (AEs) was summarized and tabulated by incidence. Pharmacokinetic parameters were calculated using Phoenix WinNonlin version 8.3.1 (Certara, Princeton, NJ, USA).

## Results

### Patients

**Fig. 1:**
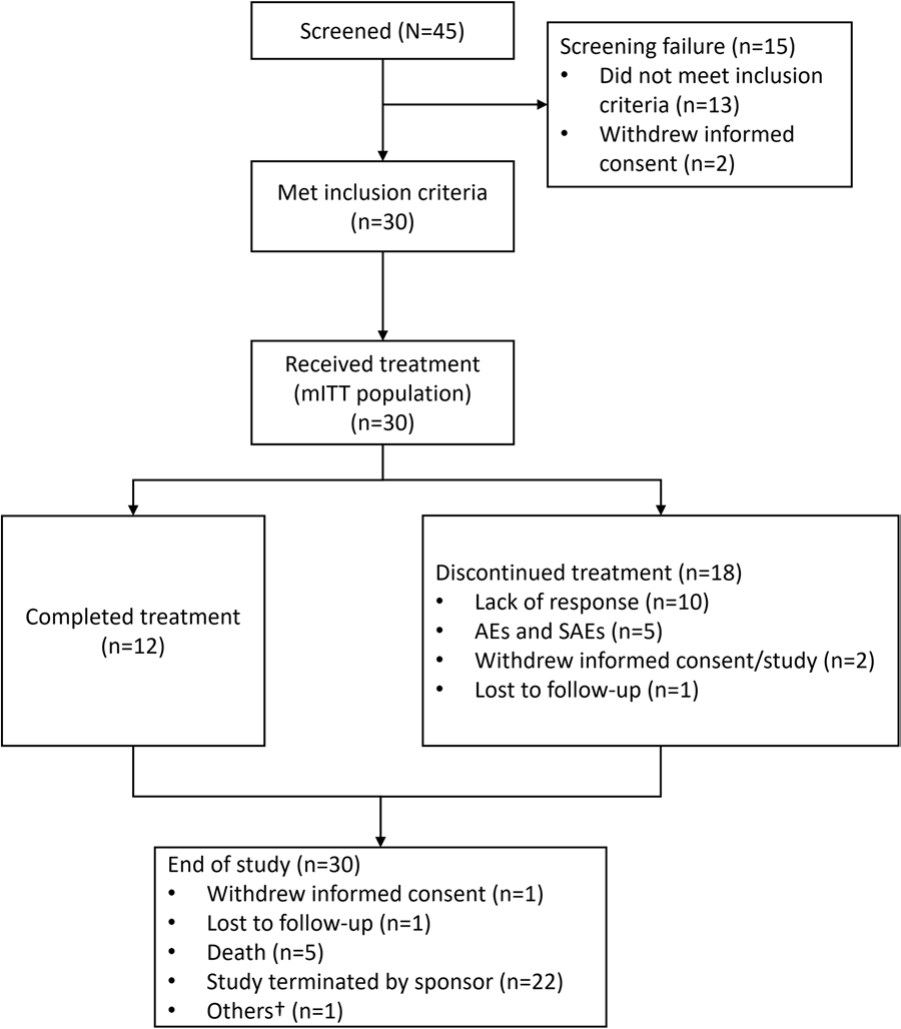
Patient disposition. †Unwilling to follow-up through phone interview

Of the 45 patients who were screened from seven study sites, 13 did not meet the inclusion criteria and two withdrew informed consent prior to the first dose. A total of 30 patients were enrolled in the study and all had at least one dose of study medication, and thus were included in the mITT population (Fig. 1). The median age of the mITT population was 30.6 (range: 18–50) years. At data cut-off (10 December 2022; 1 year after the last patient enrolled), 18 patients (60.0%) had discontinued treatment during the study due to lack of response (*n* = 10), AEs and SAEs (*n* = 5), withdrawal of consent (*n* = 2), and loss to follow-up (*n* = 1). Twelve patients (40.0%) completed the treatment at the end of the study. As of 10 December 2022, the median follow-up time was 12.9 months (range: 1.7–18.4).

Baseline demographics and cGVHD disease characteristics are shown in Table 1. Duration of cGVHD at study enrollment was 24.2 months. Of the 30 mITT patients, disease was severe at screening in 20 (66.7%) patients. The median (range) number of organs/systems involved was four (one to six) for all patients amongst whom 16 (53.3%) had at least four organs/systems involved. Mouth and eye (*n* = 22 each, 73.3%) were the most commonly involved organs in this cGVHD population. Patients received a median of three prior lines of systemic cGVHD therapy prior to the study; 27 (90.0%) and 13 (43.3%) patients had received at least two and four lines of treatment, respectively. Twenty-three (76.7%) patients were refractory to their last line of therapy prior to entering this study. All patients had received stable corticosteroid therapy for at least 2 weeks (mean prednisone dose of 0.28 mg/kg/day [range: 0.03–0.98] or equivalent at enrollment) and had disease progression or lack of response at screening.

**Table 1:** Patient baseline demographic and disease characteristics

Characteristics	All patients
**Data are *n* (%) unless stated otherwise**	**(*N* = 30)**
Age (years), median (range)	29.5 (18–50)
Sex	
Male	21 (70.0%)
Female	9 (30.0%)
Mean weight (SD), kg	56.2 (13.5)
Mean BMI (SD), kg/m^2^	19.8 (3.5)
ECOG PS	
0	9 (30.0%)
1	21 (70.0%)
Allo-HSCT	
Yes	30 (100.0%)
Indication for transplant	
Acute lymphoblastic leukemia	10 (33.3%)
Acute myeloid leukemia	8 (26.7%)
Myelodysplastic syndrome	7 (23.3%)
Chronic myelogenous leukemia	4 (13.3%)
Leukemia (other)	1 (3.3%)
Conditioning intensity	
Myeloablative	26 (86.7%)
Unknown	4 (13.3%)
Type of donor	
Related donor	27 (90.0%)
Unrelated donor	3 (10.0%)
Human leukocyte antigen matching of donor/recipient	
Matched	19 (63.3%)
Partially matched	10 (33.3%)
Unknown	1 (3.3%)
Stem cell source	
Peripheral blood stem cell	21 (70.0%)
Bone marrow	2 (6.7%)
Peripheral blood stem cell + bone marrow	6 (20.0%)
Cord blood	1 (3.3%)
Donor/recipient cytomegalovirus serological status	
+ / +	4 (13.3%)
− / −	2 (6.7%)
Unknown/ +	1 (3.3%)
Unknown/ −	16 (53.3%)
Unknown/unknown	7 (23.3%)
History of aGVHD	
Yes	17 (56.7%)
No	13 (43.3%)
Time from cGVHD diagnosis to enrollment, median (range), month	24.2 (3.4–92.9)
Number of organs involved, median (range)	4 (1–6)
Organ involvement	
1	2 (6.7%)
2	6 (20.0%)
3	6 (20.0%)
4	10 (33.3%)
5	5 (16.7%)
≥ 6	1 (3.3%)
Eyes	22 (73.3%)
Mouth	22 (73.3%)
Skin	20 (66.7%)
Lungs	13 (43.3%)
Joints/fascia	9 (30.0%)
Liver	9 (30.0%)
Esophagus	5 (16.7%)
Upper GI tract	3 (10.0%)
cGVHD severity	
Severe	20 (66.7%)
Moderate	9 (30.0%)
Mild	1 (3.3%)
Prior lines of therapies	
1	3 (10.0%)
2	8 (26.7%)
3	6 (20.0%)
4	10 (33.3%)
5	3 (10.0%)
Median, range	3 (1–5)
Refractory to the last systemic cGVHD treatment prior study enrollment	23 (76.7%)
Prior systemic cGVHD therapy type	
Prednisone/methylprednisolone	30 (100.0%)
Cyclosporine	19 (63.3%)
Tacrolimus	17 (56.7%)
Ruxolitinib	16 (53.3%)
Mycophenolate mofetil	13 (43.3%)
Methotrexate	9 (30.0%)
Sirolimus	6 (20.0%)
Ibrutinib	4 (13.3%)
Imatinib	3 (10.0%)
Azathioprine	3 (10.0%)
Rituximab	1 (3.3%)
Stem cells (unspecified)	1 (3.3%)
Zanubrutinib	1 (3.3%)
Cyclophosphamide	1 (3.3%)
TNF receptor 2-Fc fusion protein	1 (3.3%)
Concomitant systemic cGVHD medications on cycle 1 day 1 (≥ 10% patients)	
Prednisone/methylprednisolone	30 (100.0%)
Tacrolimus	13 (43.3%)
Cyclosporine	7 (23.3%)
Mycophenolate mofetil	5 (16.7%)

*aGVHD* acute graft-versus-host disease, *allo-HSCT* allogenic hematopoietic stem cell transplant, *BMI* body mass index, *cGVHD* chronic graft-versus-host disease, *ECOG PS* Eastern Cooperative Oncology Group Performance Status, *GI* gastrointestinal, *SD* standard deviation, *TNF* tumor necrosis factor

### Efficacy

**Fig. 2:**
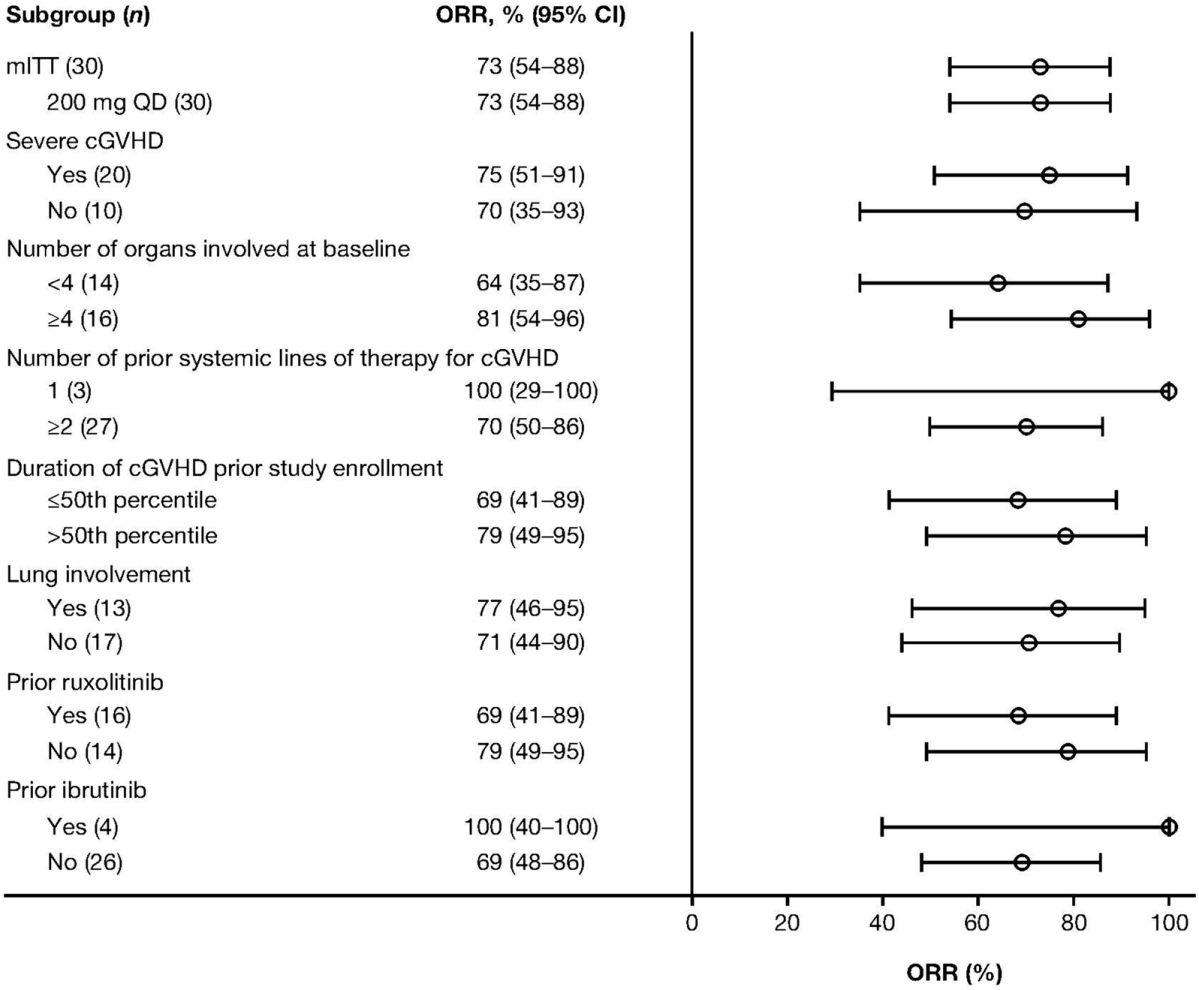
Forest plot for subgroup analyses of ORR in the mITT population. *cGVHD* chronic graft-versus-host disease, *CI* confidence interval, *mITT* modified intent-to-treat, *ORR* overall response rate, *QD* once daily

Results of the primary and secondary endpoints are summarized in Table 2. The primary efficacy endpoint ORR was 73.3% (95% CI: 54.1–87.7%), and all responses were PRs (*n* = 22). High ORR was maintained in all subgroups (Fig. 2) regardless of cGVHD severity, number of organs involved, prior number of lines of therapy, or prior ibrutinib and ruxolitinib treatment. The best response to belumosodil in each organ is listed in Additional file 1: Table S3. Responses, including CRs, were observed in all affected organs (Additional file 1: Figure S1). A total of seven patients were treated with glucocorticoids alone without any other immunosuppressive therapy at screening and during the study period, and six achieved response, with an ORR of 85.7% in this subgroup of patients. In the mITT population, ORR was observed at 40.0% in the skin, 27.3% in the eyes, 54.5% in the mouth, 60.0% in the esophagus, 66.7% in the upper gastrointestinal tract and liver, 15.4% in the lungs, and 77.8% in the joints/fascia.

**Table 2:** Response rates in the mITT population

Response Data are *n* (%) unless stated otherwise	Belumosudil 200 mg once daily (*N* = 30)
ORR	22 (73.3%)
95% CI	54.1–87.7%
PR	22 (73.3%)
ORR for responses occurring within 6 months of treatment	20 (66.7%)
95% CI	47.2–82.7%
PR	20 (66.7%)
Median DOR, weeks (95% CI)	NR (20.3–NR)
Median TTR, weeks (range)	4.3 (3.9–48.1)
Improvement from baseline based on LSS^a^	
Overall	15 (50.0%)
Responder, *n*/*N* (%)	12/22 (54.5%)
Non-responder, *n*/*N* (%)	3/8 (37.5%)
Median duration of improvement, weeks (range)	16 (4–69)
≥ 16 weeks	9 (30.0%)
≥ 24 weeks	5 (16.7%)
≥ 32 weeks	5 (16.7%)
Improvement from baseline based on LSS on two consecutive visits^a^	
Overall	10 (33.3%)
Responder, *n*/*N* (%)	9/22 (40.9%)
Non-responder, *n*/*N* (%)	1/8 (12.5%)
FFS, % (95% CI)	
Median, months	NR (7.8–NR)
6 months	73 (54–86%)
12 months	56 (37–72%)
TTNT, % (95% CI)	
Median, months	NR (8.9–NR)
6 months	77 (57–88%)
12 months	63 (43–77%)
OS, % (95% CI)	
Median, months	NR (NR–NR)
6 months	97 (79–100%)
12 months	87 (68–95%)

*CI* confidence interval, *DOR* duration of response, *FFS* failure-free-survival, *LSS* Lee symptom scale, *NR* not reached, *ORR* overall response rate, *OS* overall survival, *PR* partial response, *TTNT* time-to-next-treatment, *TTR* time-to-response

^a^Clinically meaningful improvement in cGVHD symptom burden defined as a decrease ≥ 7 points in LSS score from baseline

**Fig. 3:**
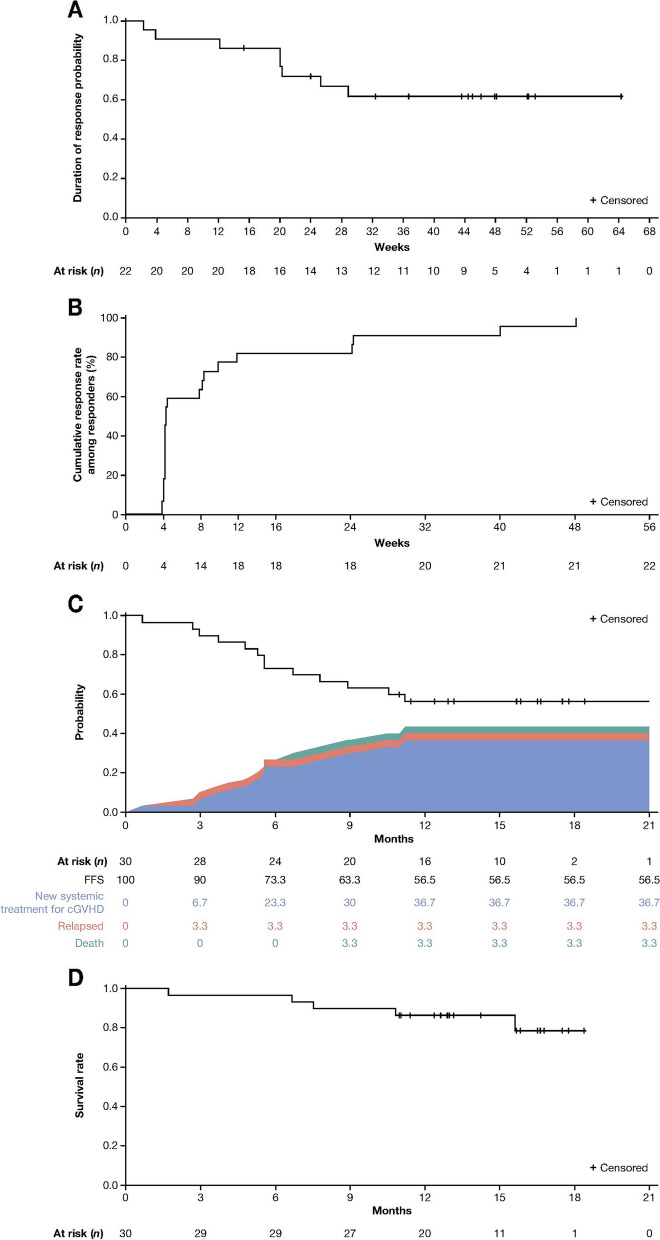
Kaplan–Meier curves for (**a**) DOR among responders, (**b**) time to response among responders, (**c**) estimated FFS in the mITT population, and (**d**) OS in the mITT population. DOR was defined as the time from the first assessment of CR or PR to the first detection of a lack of response. If the patient experienced progression from CR or PR to a lack of response for more than one time, the cumulative DOR was recorded. *CR* complete response, *DOR* duration of response, *FSS* failure-free survival, *mITT* modified intent-to-treat, *OS* overall survival, *PR* partial response

Median DOR among responders was not reached (NR) (Fig. 3a). Median TTR was 4.3 weeks (range: 3.9–48.1); 17 patients achieved a response by week 10 and an additional five patients responded to therapy afterwards (Fig. 3b). The median FFS rate was NR; 6- and 12-month FFS rates were 73.0% (95% CI: 54.0–86.0%) and 56% (95% CI: 37.0–72.0%), respectively (Fig. 3c). The most common failure event was initiating new systemic therapy (*n* = 11, 36.7%). The median TTNT was NR; at 6 and 12 months, 77.0% (95% CI: 57.0–88.0%) and 63.0% (95% CI: 43.0–77.0%) of patients had not yet started next treatment, respectively. Median OS was NR; 6- and 12-month OS rates were 97.0% (95% CI: 79.0–100.0%) and 87% (95% CI: 68.0–95.0%), respectively (Fig. 3d).

Clinically meaningful improvement (reduction in LSS by ≥ 7 points from baseline) was observed in 15 (50.0%) patients (12 responders and 3 non-responders) with a mean duration of 16 weeks (range: 4–69), and 10 (33.3%) patients (9 responders and 1 non-responder) reported clinical improvement in two consecutive visits. All 30 patients were using stable dose corticosteroid at study enrollment, and corticosteroid dose was reduced in 17 (56.7%) patients amongst whom eight patients discontinued corticosteroids for ≥ 28 days during the study. Median corticosteroid dose reduction in the overall population was 33.3% (Additional file 1: Table S4). A higher proportion of patients with corticosteroid dose reduction was reported among responders than non-responders (63.6% versus 37.5%). Among those who were concomitantly receiving CNIs (*n* = 20) at study enrollment, CNI dose reduction was seen in seven (35.0%) patients and discontinued in three patients (15.0%) (Additional file 1: Table S4).

Changes in the PROMIS global health subscores for physical and mental functioning were assessed. Twelve (41.4%) patients in the overall population (11/22 responders and 1/7 non-responders) had an increase in PROMIS physical health score of ≥ 4.7 points from baseline. Fifteen (51.7%) patients in the overall population (13/22 responders and 2/7 non-responders) had an increase in PROMIS mental health score of ≥ 4.7 points from baseline.

### Safety

Median duration of drug exposure was 10.3 months (range: 0.5–18.4); median relative dose intensity was 99.5% (range: 91.1–100.0%) and 28 (93.3%) patients received a median relative dose of > 95%. A summary of the AEs is shown in Table 3. A total of 29 (96.7%) patients reported at least one TEAE during the study; drug-related TEAEs were observed in 19 (63.3%) patients and11 (36.7%) patients had SAEs. Grade ≥ 3 TEAEs were reported in 11 (36.7%) patients. The most common (≥ 5% of patients) grade ≥ 3 TEAE was pneumonia (*n* = 5, 16.7%). One patient experienced clinically significant cytopenia events that were deemed unrelated to belumosudil. Cytomegalovirus infection was reported in one patient (3.3%), possibly unrelated to belumosudil.

**Table 3:** Overview of AEs

AE Data are *n* (%) unless stated otherwise	Belumosudil 200 mg once daily (*N* = 30)
Any AEs	29 (96.7%)
Grade ≥ 3 AEs	11 (36.7%)
Drug-related AEs	19 (63.3%)
SAEs	11 (36.7%)
Drug-related SAEs	4 (13.3%)
AEs leading to dose reduction	1 (3.3%)
AEs leading to dose interruption	2 (6.7%)
AEs leading to treatment discontinuation	5 (16.7%)
AEs leading to death	4 (13.3%)
All-grade TEAEs (≥ 10% of patients)	
Respiratory tract infection^a^	14 (46.7%)
Sinus tachycardia	11 (36.7%)
Pneumonia	7 (23.3%)
Hypoproteinemia^b^	6 (20.0%)
Hyperuricemia	5 (16.7%)
Liver injury	5 (16.7%)
Alanine aminotransferase increased	3 (10.0%)
Blood creatinine increased	3 (10.0%)
Blood glucose increased	3 (10.0%)
Hypertension	3 (10.0%)
Nausea	3 (10.0%)
Vomiting	3 (10.0%)
Grade ≥ 3 TEAEs (≥ 5% of patients)	
Pneumonia	5 (16.7%)

*AE* adverse event, *SAE* serious adverse event, *TEAE* treatment-emergent adverse event

^a^Respiratory tract infection includes AE of upper respiratory tract infection and respiratory tract infection

^b^Hypoproteinemia includes AE of hypoalbuminemia and hypoproteinemia

The most common (≥ 5% of patients) drug-related TEAEs were sinus tachycardia (*n* = 9, 30.0%), upper respiratory tract infection (*n* = 4, 13.3%), alanine aminotransferase increased (*n* = 2, 6.7%), liver injury (*n* = 2, 6.7%), blood pressure increased (*n* = 2, 6.7%), and hypertension (*n* = 2, 6.7%). Four (13.3%) patients experienced at least one grade ≥ 3 drug-related TEAE including alanine aminotransferase increased, lymphocyte count decreased, pneumonia, skin bacterial infection, and muscle spasms (each *n* = 1, 3.3%).

Dose reduction was reported in one (3.3%) patient due to alanine aminotransferase increased, which was deemed drug-related by the investigator. Five (16.7%) patients who discontinued treatment due to pneumonia (*n* = 2, 6.7%), myocarditis, leukemia recurrent, and skin bacterial infection (*n* = 1 each, 3.3%), and two (6.7%) patients who had pneumonia and skin bacterial infection (*n* = 1 each, 3.3%) were likely to be drug related.

The study reported five deaths amongst which four were due to SAEs (fulminant myocarditis, lung infection, pneumonia, and leukemia, *n* = 1 each) with only one (pneumonia) possibly related to belumosudil. The patient with fulminant myocarditis died within 30 days after receiving the last dose of treatment. The other four deaths occurred more than 30 days after the last dose of study treatment.

### Pharmacokinetics

The first 12 consented patients were included in the pharmacokinetics subgroup with an extensive blood sampling schedule (Additional file 1: Table S2). The plasma concentration time curves of belumosudil at first dose and multiple doses among patients on the extensive blood sampling schedule are presented in Additional file 1: Figure S2. Plasma concentration of belumosudil increased quickly after administration, peaking between 2 and 4 h, before declining gradually over 24 h. After the first administration of belumosudil 200 mg once daily, the median T_max_ was 2.5 h (range: 1.4–5.8), mean T_1/2_ 3.8 h, mean C_max_ 4284.6 ng/mL, mean AUC_0-t_ 22,347.5 h*ng/mL, and mean AUC_0-inf_ 22,844.5 h*ng/mL. The average plasma concentration time curve after multiple administrations was similar to that of the first administration. There was also no accumulation of belumosudil after 28 days of continuous administration. The other pharmacokinetic parameters are presented in Additional file 1: Table S5.

## Discussion

This phase II study was the first belumosudil clinical trial in patients with cGVHD in China. The primary efficacy endpoint ORR was 73.3% (95% CI: 54.1–87.7%), which is highly consistent with that in the Western population. Given that two-thirds (66.7%) of the patients had severe cGVHD, and more than half (53.3%) had at least four organs involved, the treatment effect was clinically meaningful in this cGVHD population whose human leukocyte antigen matching was 63.3%. Treatment response was gradual (median TTR: 4.3 weeks) with nearly one-quarter of responders occurring at 20 weeks or beyond, but responses were durable (median DOR, NR). Despite the small sample size, CR (defined as resolution of all manifestations related to cGVHD) was observed in all organs, and response rates by involved organ were consistent across all subgroups as well as with ORR. Such treatment effect was considered robust given that the certain clinical manifestations of cGVHD in some organs such as the eyes (severe ocular sicca), lungs (bronchiolitis obliterans syndrome), and skin (fasciitis or cutaneous sclerosis), or when fibrosis is involved, could be difficult to treat [5].

Achieving response or disease control in patients with cGVHD could potentially translate to an improvement in quality of life and survival. The high ORR among patients with at least four organs involved and severe cGVHD suggested that belumosudil can benefit patients with severe disease. On the contrary, in the ROCKstar trial, ORR was numerically lower in patients with at least four organs involved compared with those with less than four organs involved (72.0% vs 80.0%) [10]. In addition, belumosudil achieved clinically meaningful responses in LSS score. Twelve patients among the 22 responders (54.5%) reported improvement in LSS score from baseline, five of whom achieved ≥ 32 weeks of symptom improvement, signaling improvement of physical and social functions, and hence quality of life [13]. The use of validated scales, such as LSS, to demonstrate improvement in patient-reported symptoms advocated by the NIH is an important part of measuring efficacy especially for novel therapeutic agents [12]. The corticosteroid-sparing effect of belumosudil was equally clinically meaningful as long-term use of corticosteroids can result in many comorbidities and other complications. It was observed that among the responders, 14 (63.6%) patients achieved corticosteroid reduction with a median reduction of 41.7% and six (27.3%) patients even discontinued corticosteroids. Non-responders also experienced clinical benefits as evidenced by the improvement in LSS score (*n* = 3, 37.5%) and corticosteroid reduction (*n* = 3, 37.5%).

Due to the possible heterogeneity of study populations, inter-study comparisons should be interpreted with caution. Nevertheless, disease severity and prior treatment were very similar between this study and the ROCKstar trial (i.e., majority of patients had severe cGVHD and > 40% patients had at least four organs involved). Data from this study were generally consistent with those of the ROCKstar trial where half of the patients who were treated with belumosudil received a dose of 200 mg once daily [10]. Like in this study, CR was also achieved in all organs involved in the ROCKstar trial [10]. The response rate in the lungs was not as high as that reported in the ROCKstar trial (15.4% versus 29.0%) [10], possibly due to the small number of patients included in the subgroup. Fibrotic changes in the lungs manifested as bronchiolitis obliterans can be difficult to treat hampering lung function improvement [5]. In this study, patients with lung involvement at baseline were more severe as compared with those in the ROCKstar trial: a higher proportion of patients had NIH lung symptom score of 2 compared with the ROCKstar trial (77% versus 38%) [10]. The baseline severity difference between the two studies could be another plausible explanation for the difference in response. However, this was higher than the 8.6% response rate in the lungs reported in the REACH3 study with ruxolitinib [14].

Apart from the ROCKstar trial, there was an earlier phase IIa, dose-finding study of belumosudil in cGVHD patients. Belumosudil 200 mg once daily demonstrated an ORR of 65%, and consistent responses were achieved in patients with severe cGVHD (ORR, 67%) or at least four organs involvement (ORR, 50%) [15]. Taking efficacy data as a totality, belumosudil treatment for cGVHD has shown consistent outcomes regardless of ethnicity, age, prior treatment, or disease severity. Furthermore, belumosudil treatment for cGVHD did not negatively impact long-term survival of post-transplant patients and neither did it show poorer outcome in older patients with cGVHD [16, 17]. The 1-year survival rate of 87% with belumosudil in patients who failed prior therapy was comparable to ruxolitinib (81.4%) and ibrutinib (24-month OS, 71%) [14, 18], suggesting that belumosudil did not have a detrimental effect on the long-term efficacy of allo-HSCT.

Belumosudil was well tolerated with the majority of TEAEs being mild to moderate in severity. There were no new safety signals compared with earlier studies [10, 15]. No patients reported AEs of secondary malignancies during the study, and only one cytomegalovirus infection was reported. Treatment discontinuation (*n* = 5, 16.7%), dose reduction (*n* = 1, 3.3%), and interruption (*n* = 2, 6.7%) rates due to TEAEs were low. This meant that patients could remain on therapy for a long time to achieve or maintain clinically meaningful treatment outcomes. Treatment resulted in a large proportion of patients reducing corticosteroid or CNI use, and therefore improved overall safety profile and quality of life. We reported that 11 patients (36.7%) had SAEs, which was similar to the ROCKStar trial (38.0%).

Significant toxicities, such as bone loss, metabolic disorders, and infection complications, are typically associated with long-term corticosteroid use [5]. Steroid-free options such as ibrutinib and ruxolitinib are other novel therapies that have demonstrated clinical efficacy in cGVHD; however, cross trial comparisons should be interpreted with caution given the differences in study designs and patient population. In the REACH3 study (329 patients), the best overall response at week 24 for ruxolitinib was 76.4% compared with 60.4% in the control arm [14]. All patients were glucocorticoid-refractory or -dependent and had previously received one or two lines of systemic treatment for cGVHD. Twelve patients aged between 12–17 years old were recruited in the REACH3 study, and the median age of 49.0 years was higher than that in our study. In the comparator arm of the study, patients were allowed to receive extracorporeal photopheresis, mycophenolate mofetil, imatinib, and ibrutinib, among other treatments [14]. Elsewhere, the benefit of ibrutinib in cGVHD was demonstrated in a phase Ib/II, open-label study (42 patients), with a best overall response of 67.0% and alleviation of symptoms [19]. All patients were steroid-dependent or -refractory and had received up to three previous treatment regimens for cGVHD. The median age of the patients was 56.0 years old and concomitant use of immunosuppressive agents was also allowed [19]. Hematologic side effects, such as thrombocytopenia, anemia, and neutropenia, are commonly associated with ruxolitinib due to its myelosuppressive mechanism and, therefore, often resulted in treatment interruption or discontinuation [14]; while infections and cardiac effects (atrial fibrillation and cardiac arrest) are known safety risks associated with ibrutinib treatment that led to high discontinuation rates of approximately 23–33% [19, 20]. Both agents are not yet approved for cGVHD in China. Belumosudil’s safety profile is consistent to that of post–allo-HSCT patients living with cGVHD in general, so the drug can provide a valuable treatment option to patients who have failed corticosteroid therapy.

Another important aspect of our study was to examine the pharmacokinetic profile of belumosudil in Chinese patients. Due to limited pharmacokinetic data from the ROCKstar trial, it is difficult to compare the pharmacokinetic parameters directly between Chinese and Caucasian/Western cGVHD patients.

There are several limitations to this study. First, this was not a randomized controlled study, which means that it is difficult to objectively compare belumosudil treatment effect against the physician’s choice or to quantitively assess treatment-related AEs. Nevertheless, the design and conduct of the study was ethical as there are no approved treatments for cGVHD in the second-line setting in China. In addition, with a limited sample size, subgroup analyses should be interpreted with caution. We did not compare the statistical significance of clinical outcomes between responders and non-responders. It was also not clear whether improvements in other clinical outcomes among non-responders could translate to survival benefits. Our subgroup analysis also showed that all patients who received prior ibrutinib (*n* = 4) achieved 100% ORR. While the sample size was too small to make a definitive assumption, this suggests that optimal treatment sequencing to improve clinical outcomes warrants further investigation. Another approach to improve response rates and overcome the development of resistance against belumosudil is to combine this new agent with other therapies with a different modality to harness synergy from the different agents [21]. However, such an approach has been confined mostly to retrospective studies; this warrants further investigation of both the combination of belumosudil in the clinical trial setting and for any potential drug interaction with other agents.

## Conclusions

In conclusion, given that there is currently no standard second-line treatment for cGVHD in China, belumosudil could fill the gap of unmet medical need as a valuable treatment option that has been demonstrated to be effective and safe in patients who failed corticosteroid treatment in China.

### Supplementary Information

**Additional file 1: Table S1.** Inclusion and exclusion criteria. **Table S2.** Blood sampling schedule. **Table S3.** Best response to belumosudil in each organ. **Table S4.** Corticosteroid and calcineurin inhibitor dose reduction. **Table S5.** A summary of pharmacokinetic parameters after first and multiple doses. **Figure S1.** ORR by organ type in the mITT population. **Figure S2.** Plasma concentration time curve after (a) first dose and (b) multiple doses.

## Data Availability

The datasets used and/or analyzed during the current study are included in this article or as supplementary material in Additional file 1. Any additional data requests are available from the corresponding author upon reasonable request.

## Abbreviations

AE: Adverse event
aGVHD: Acute graft-versus-host disease
allo-HSCT: Allogenic hematopoietic stem cell transplant
AUC_0-inf_: Area under the curve from zero to time infinity
AUC_0-t_: Area under the curve up to the last quantifiable time-point
BMI: Body mass index
cGVHD: Chronic graft-versus-host disease
CI: Confidence interval
C_max_: Maximum serum concentration
CNI: Calcineurin inhibitor
CR: Complete response
DOR: Duration of response
ECOG PS: Eastern Cooperative Oncology Group Performance Status
FFS: Failure-free survival
GI: Gastrointestinal
LSS: Lee Symptom Scale
mMITT: Modified intent-to-treat
NR: Not reached
ORR: Overall response rate
OS: Overall survival
PR: Partial response
QD: Once daily
ROCK2: Rho-associated, coiled-coil-containing protein kinase 2
SAE: Serious adverse event
SD: Standard deviation
TEAE: Treatment-emergent adverse event
T_max_: Time of peak plasma concentration
TNF: Tumor necrosis factor
Treg: Regulatory T
TTNT: Time-to-next-treatment
TTR: Time to response
T_1/2_: Half-life
