# Supplementary material for: A phase II study of belumosudil for chronic graft-versus-host disease in patients who failed at least one line of systemic therapy in China
Source: BMC Med. 2024 Mar 26;22:142. doi: 10.1186/s12916-024-03348-5 (PMC10964632; doi:10.1186/s12916-024-03348-5)

## Table S1. Inclusion and exclusion criteria

| **Inclusion criteria**  Patients who met **all** of the following conditions could be enrolled in the study:   1. Male or female patients aged ≥ 18 years who have had allogeneic hematopoietic stem cell transplant (allo-HSCT). 2. Have persistent cGVHD manifestations and systemic therapy is indicated. 3. Previously received at least 1 and not more than 5 lines of systemic therapy for cGVHD. 4. Receiving glucocorticoid therapy with a stable dose over the 2 weeks prior to screening; or 4 weeks of prednisone or equivalent doses of other corticosteroids at doses > 0.5 mg/kg/day with persistent manifestations of cGVHD and no improvement; or 2 attempts to reduce the hormone to a lower dose level fail and the prednisone dose still needs to be increased to > 0.25 mg/kg/day or an equivalent dose. 5. Eastern Cooperative Oncology Group (ECOG) performance status (PS) score: 0 to 1. 6. Life expectancy of more than 12 months.   General criteria   1. Female patients of childbearing potential have a negative serum pregnancy test at screening. Females of childbearing potential are defined as sexually mature females without prior hysterectomy or who have had any evidence of menses in the past 12 months. However, females who have been amenorrheic for 12 or more months are still considered to be of childbearing potential if the amenorrhea is possibly due to prior chemotherapy, anti-estrogens, or ovarian suppression. 2. Sexually active females of childbearing potential enrolled in the study must agree to use two forms of accepted methods of contraception during the course of the study and for 3 months after their last dose of study drug. Effective birth control includes:  - Intra-uterine contraceptive device plus 1 barrier method; - Stable doses of hormonal contraception (e.g., oral, injectable, subcutaneously implanted, transdermal) for at least 3 months plus 1 barrier method; - Two barrier methods. Effective barrier methods are male or female condoms, diaphragms, and spermicides (creams or gels that contain a chemical to kill sperm); or - A vasectomized partner.  1. For male patients who are sexually active and who are partners of females of childbearing potential: must agree to use 2 recognized methods of contraception during the treatment period and for 3 months after the last dose of study drug (see criterion 8 above). 2. Subject (or the subject's legally authorized representative) must be fully informed of the study contents and is able to provide written ICF prior to any study-specific procedures, and is willing to follow the treatment regimen and visit schedule. |
| --- |
| **Exclusion criteria**  Patients who met **any** of the following conditions would be excluded from the study:   1. Received a systemic investigational cGVHD treatment within 28 days of study entry, but prior treatment is allowed with a washout of at least 28 days or 5 half-lives. [Note: Corticosteroids, CNIs, sirolimus, mycophenolate mofetil (MMF), methotrexate, azathioprine, and *in vitro* photochemotherapy (ECP) are acceptable and patients must have been on a stable dose/regimen of these for at least 2 weeks prior to screening]. 2. Recurrence of hematologic tumor (according to criteria for recurrence of the corresponding primary hematologic tumor) or post-transplant lymphoproliferative disease at screening. 3. Current treatment with ibrutinib (except for ibrutinib with a washout of at least 28 days prior to the first dose of the investigational product).   Laboratory Tests   1. Absolute neutrophil count (ANC) < 1.5 × 10^9^/L. 2. Platelet count < 50 × 10^9^/L. 3. Alanine aminotransferase (ALT) > 3 × upper limit of normal (ULN), aspartate aminotransferase (AST) > 3 × ULN 4. Total bilirubin (TBIL) > 1.5 × ULN. 5. Creatinine clearance (CrCl) <60 mL/min (Cockcroft-Gault formula).   General Criteria   1. Pregnant or lactating women. 2. History of severe illness, or other evidence of severe illness, or any other conditions that would make the subject, in the opinion of the investigator, unsuitable for the study  - History of severe [New York Heart Association (NYHA) functional class III or IV] cardiovascular disorder, including but not limited to ventricular arrhythmias requiring clinical intervention, uncontrolled hypertension (systolic blood pressure ≥ 160 mmHg and/or diastolic blood pressure ≥100 mmHg); unstable angina pectoris, acute coronary syndrome, congestive cardiac failure, stroke, or other ≥ Grade 3 cardiac events within 6 months prior to enrollment; and NYHA functional class ≥ II or left ventricular ejection fraction (LVEF) < 50% by cardiac ultrasound at screening. - Inability to take oral medications, severe (NCI CTCAE v5.0 ≥ Grade 3) chronic gastrointestinal dysfunction, malabsorption syndrome, or any other condition that affects gastrointestinal absorption. - History of clear neurological or mental disorders (including epilepsy or dementia), current mental disorders, or poor compliance that rendered the subject ineligible for participation in the study as judged by the investigator. - History of other serious (NCI CTCAE v5.0≥ Grade 3) systemic disease that rendered the subject ineligible for participation in the clinical trial as judged by the investigator.  1. Known active hepatitis B virus (HBV) or hepatitis C virus (HCV) infection, or human immunodeficiency virus (HIV) infection. [Note: Active HBV infection is defined as positive for serum hepatitis B virus surface antigen (HBsAg) and/or hepatitis B virus e antigen (HBeAg), or HBV-DNA; patients with positive hepatitis B virus core antibody (anti-HBc) should be confirmed for HBV-DNA, and patients who are confirmed to be negative for HBV-DNA could be enrolled; active HCV infection is defined as positive for HCV-RNA, and patients who are positive for hepatitis C virus antibody (HCV-Ab) could only be enrolled after confirmed to be negative for HCV-RNA. Positive is defined as ＞ the ULN]. 2. Diagnosed with another primary malignancy (other than malignancy for which allo-HSCT was performed) within 3 years of enrollment, with the exception of:  - Completely resected basal cell or squamous cell carcinoma of the skin; - Surgically cured carcinoma in situ of the cervix; - Resected breast ductal carcinoma in situ; - Prostate cancer with Gleason score < 6 and stable prostate-specific antigen (PSA) over 12 months.  1. Known allergy to the active ingredient or excipients of the investigational product, or any other selective ROCK2 inhibitors. 2. Patients requiring long-term proton pump inhibitors (e.g., rabeprazole, omeprazole) or CYP3A4 inducers (e.g., rifampin, phenobarbital). 3. Prolongation of QT interval corrected by the Fridericia's formula (QTcF) of > 450 ms for males and > 470 ms for females at screening. 4. Known alcohol or drug dependence. 5. Forced expiratory volume in 1 second (FEV1) ≤39% or pulmonary function score of 3 at screening. 6. Treatment with any investigational agent, device, or procedure within 28 days (or 5 half-lives, whichever is longer) prior to enrollment. 7. Patients considered unlikely to adhere to treatment and follow protocol in the opinion of the investigator. |

## Table S2. Blood sampling schedule

| **Cycle** | **Day** | **Blood collection time points** |
| --- | --- | --- |
| **Extensive blood sampling (n=12)** | | |
| 1 | 1 | Before receiving belumosudil; after receiving belumosudil at 1, 1.5, 2, 3, 4, 6, 8, and 12 hours |
|  | 2 | Before receiving belumosudil |
|  | 15 | Before receiving belumosudil |
| 2 (or when withdrawing from the study early) | 1 | Before receiving belumosudil; after receiving belumosudil at 1, 1.5, 2, 3, 4, 6, 8, and 12 hours |
|  | 2 | Before receiving belumosudil |
| 3 (or when withdrawing from the study early) | 1 | Before receiving belumosudil |
| 4 (or when withdrawing from the study early) | 1 | Before receiving belumosudil; after receiving belumosudil at 1.5 and 4 hours |
| **Limited blood sampling (all remaining patients)** | | |
| 2 (or when withdrawing from the study early, except for those who were in the extensive blood sampling group) | 1 | Before receiving belumosudil; after receiving belumosudil at 1.5 and 4 hours |
| 4 (or when withdrawing from the study early) | 1 | Before receiving belumosudil; after receiving belumosudil at 1.5 and 4 hours |

## Table S3. Best response to belumosudil in each organ

|  | **mITT population** | **Response population** |
| --- | --- | --- |
|  | (N=30) | (N=22) |
| Skin，Nx | 20 | 15 |
| Complete Response, n (%) | 3 (15.0%) | 3 (20.0%) |
| Partial Response, n (%) | 5 (25.0%) | 5 (33.3%) |
| Overall Response (PR+CR), n (%) | 8 (40.0%) | 8 (53.3%) |
|  |  |  |
| Eyes，Nx | 22 | 15 |
| Complete Response, n (%) | 5 (22.7%) | 4 (26.7%) |
| Partial Response, n (%) | 1 (4.5%) | 1 (6.7%) |
| Overall Response (PR+CR), n (%) | 6 (27.3%) | 5 (33.3%) |
|  |  |  |
| Mouth，Nx | 22 | 16 |
| Complete Response, n (%) | 6 (27.3%) | 5 (31.3%) |
| Partial Response, n (%) | 6 (27.3%) | 6 (37.5%) |
| Overall Response (PR+CR), n (%) | 12 (54.5%) | 11 (68.8%) |
|  |  |  |
| Esophagus，Nx | 5 | 3 |
| Complete Response, n (%) | 2 (40.0%) | 2 (66.7%) |
| Partial Response, n (%) | 1 (20.0%) | 1 (33.3%) |
| Overall Response (PR+CR), n (%) | 3 (60.0%) | 3 (100.0%) |
|  |  |  |
| Upper gastrointestinal tract，Nx | 3 | 3 |
| Complete Response, n (%) | 2 (66.7%) | 2 (66.7%) |
| Partial Response, n (%) | 0 | 0 |
| Overall Response (PR+CR), n (%) | 2 (66.7%) | 2 (66.7%) |
|  |  |  |
| Liver，Nx | 9 | 8 |
| Complete Response, n (%) | 1 (11.1%) | 1 (12.5%) |
| Partial Response, n (%) | 5 (55.6%) | 5 (62.5%) |
| Overall Response (PR+CR), n (%) | 6 (66.7%) | 6 (75.0%) |
|  |  |  |
| Lung，Nx | 13 | 10 |
| Complete Response, n (%) | 1 (7.7%) | 1 (10.0%) |
| Partial Response, n (%) | 1 (7.7%) | 1 (10.0%) |
| Overall Response (PR+CR), n (%) | 2 (15.4%) | 2 (20.0%) |
|  |  |  |
| Joints/fascia，Nx | 9 | 6 |
| Complete Response, n (%) | 2 (22.2%) | 2 (33.3%) |
| Partial Response, n (%) | 5 (55.6%) | 4 (66.7%) |
| Overall Response (PR+CR), n (%) | 7 (77.8%) | 6 (100.0%) |
|  |  |  |
| Overall severity level，Nx | 30 | 22 |
| Complete Response, n (%) | 1 (3.3%) | 1 (4.5%) |
| Partial Response, n (%) | 8 (26.7%) | 8 (36.4%) |
| Overall Response (PR+CR), n (%) | 9 (30.0%) | 9 (40.9%) |

Nx, the number of subjects in the mITT population/response population who had involvement of the organ at baseline and the number of subjects who had an overall severity rating assessment at baseline.

Percentage of each organ was calculated using Nx as the denominator.

## Table S4. Corticosteroid and calcineurin inhibitor dose reduction

| **Response**  Data are n (%) unless stated otherwise | **Belumosudil 200 mg QD (N=30)** |
| --- | --- |
| **Proportion of patients with corticosteroid dose reduction** |  |
| Overall | 17 (56.7%) |
| Responder, n/N (%) | 14/22 (63.6%) |
| Nonresponder, n/N (%) | 3/8 (37.5%) |
| **Median change in corticosteroid dose from baseline, % (range)** |  |
| Overall | −33.3 (−100.0 to 700.0) |
| Responder | −41.7 (−100.0 to 0.0) |
| Nonresponder | 0.0 (−100.0 to 700.0) |
| **Proportion of patients who discontinued corticosteroid for at least 28 days** |  |
| Overall | 8/30 (26.7%) |
| Responder, n/N (%) | 6/22 (27.3%) |
| Nonresponder, n/N (%) | 2/8 (25.0%) |
| **Proportion of patients on CNI** | 20 (66.7%) |
| CNI dose reduction, n/N (%) | 7/20 (35.0%) |
| CNI discontinuation, n/N (%) | 3/20 (15.0%) |

## Table S5. A summary of pharmacokinetic parameters after first and multiple doses

| **Pharmacokinetic parameters** | **First dose (N=12)** | **Multiple doses (N=12)** |
| --- | --- | --- |
| Mean C_max_, ng/mL | 4284.59 | 4673.43 |
| Median T_max_, h (range) | 2.47 (1.42–5.83) | 3.03 (1.92–5.88) |
| Mean AUC_0-t_, h*ng/mL | 22347.47 | 28938.55 |
| Mean AUC_0-inf_, h*ng/mL | 22844.51 | 29923.26 |
| Mean T_1/2_, h | 3.79 | 4.54 |
| Mean AUC_tau_, h*ng/mL | 22387.02 | 29042.16 |

AUC_0-inf_, area under the concentration time-curves from time zero to infinity; AUC_tau_, area under plasma concentration-time curve overdosing interval AUC_0-t_, area under the concentration-time curve from dosing (time 0) to the time of the last measured concentration; C_max_, maximum plasma concentration; T_max_, time of peak plasma concentration; T_1/2_, time required for plasma concentration of a drug to decrease by 50%.

## Figure S1 .ORR by organ type in the mITT population

CR, complete response; mITT, modified intent-to-treat; ORR, overall response rate; PR, partial response.

## Figure S2. Plasma concentration time curve after (a) first dose and (b) multiple doses

(a)


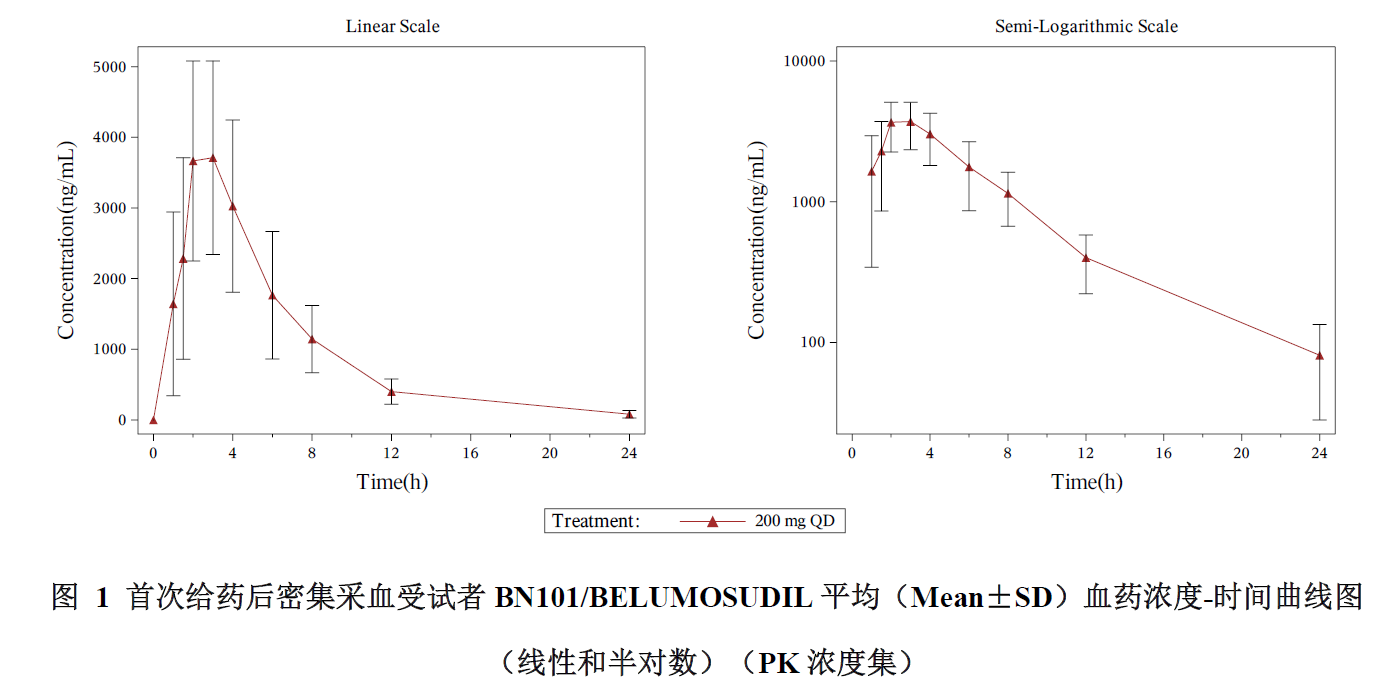


(b)


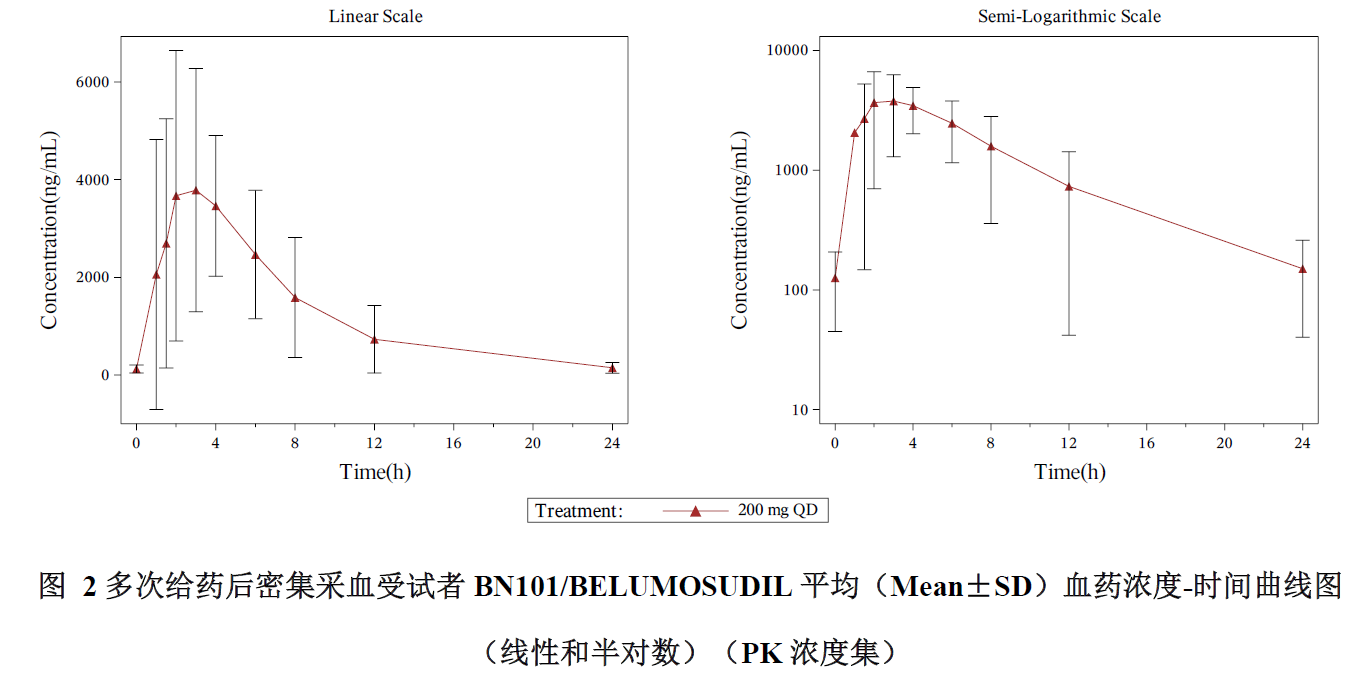

Supplement: Supplementary file 1 — Additional file 1: Table S1. Inclusion and exclusion criteria. Table S2. Blood sampling schedule. Table S3. Best response to belumosudil in each organ. Table S4. Corticosteroid and calcineurin inhibitor dose reduction. Table S5. A summary of pharmacokinetic parameters after first and multiple doses. Figure S1. ORR by organ type in the mITT population. Figure S2. Plasma concentration time curve after (a) first dose and (b) multiple doses. [file 12916_2024_3348_MOESM1_ESM.docx]
